# Fixation of Light Weight Polypropylene Mesh with n-Butyl-2-cyanocrylate in Pelvic Floor Surgery: Experimental Design Approach in Sheep for Effectiveness Evaluation

**DOI:** 10.1155/2015/737683

**Published:** 2015-06-28

**Authors:** Sandra Barbosa, Tania Nieves, Félix García, Eva Cepeda, Xavier Moll, Alberto Marco, Christine Weis, Pau Turon, Patri Vergara

**Affiliations:** ^1^Department of Cell Biology, Physiology and Immunology, Facultat de Veterinària, Universitat Autònoma de Barcelona, Bellaterra, 08153 Barcelona, Spain; ^2^Integrated Services of Laboratory Animals, Universitat Autònoma de Barcelona, Bellaterra, 08153 Barcelona, Spain; ^3^B. Braun Surgical S.A., Rubi, 08191 Barcelona, Spain; ^4^Department of Animal Medicine and Surgery, Facultat de Veterinària, Universitat Autònoma de Barcelona, Bellaterra, 08153 Barcelona, Spain; ^5^Department of Animal Health and Anatomy, Facultat de Veterinària, Universitat Autònoma de Barcelona, Bellaterra, 08153 Barcelona, Spain

## Abstract

*Objective*. The aim of this study was to find a proper experimental design and to evaluate n-butyl-2-cyanoacrylate (Histoacryl) as a fixation method for a light-weight and large pore PP mesh (*Synthetic PP Mesh-1*) using the sheep as an animal model. *Methods*. Posterior vaginal implantation by means of episiotomy was used to implant 8 ewes which were evaluated macroscopically and histologically at 3 months (*n* = 4) and 6 months (*n* = 4) post-surgery. In previous pilot studies anterior vaginal implantation was evaluated, as well as different synthetic mesh materials, sizes and fixation methods (*n* = 1 to 3) during three weeks. In all cases a clinical evaluation of the animal was performed. *Results*. A reduction in the mesh size (*Synthetic PP Mesh-1*) together with precise application of the surgical glue Histoacryl to fix the mesh yielded significantly better histocompatibility results (*P* < 0.01) compared to larger size or other fixation methods. *Conclusion*. The combination of *Synthetic PP Mesh-1* with Histoacryl offered a high degree of graft integration without vaginal ulceration and a minimal foreign body reaction, being the sheep a proper animal model to test these types of medical devices.

## 1. Introduction

Pelvic organ prolapse (POP) is a common occurrence that has become more prevalent in view of the aging population. It is not a life threating condition and death rate is almost nonexistent but it substantially affects the quality of life. The normal occurrence of pelvic organ support defects in women has still not been properly investigated. However, recent studies have described a POP prevalence of 30–50% diagnosticated by gynecologic examination in women between 45 and 85 years of age, with prevalence of symptomatology reported by the patient ranging from 4 to 12% [1, 2].

Vaginal synthetic meshes help reinforce pelvic floor tissue and were proposed for use in reconstructive pelvic surgery mainly to reduce the high rate of recurrences of patients operated with native-tissue methods [3–5]. Furthermore, the use of prostheses allows for greater procedure standardization, shorter surgery duration, and faster postoperative recovery.

However, transvaginal mesh surgery has been associated with specific complications related to mesh quality, surgical technique, and surgeon's experience with the occurrence of mesh exposure, in combination with dyspareunia, being the most frequent complication [6, 7]. For this reason, the US Food and Drug Administration (FDA) has already issued two Public Health Notifications (2008 and 2011) regarding the potential for serious complications associated with transvaginal placement of surgical mesh in repair of POP [8]. In Europe, the Scientific Committee on Emerging and Newly Identified Health Risks (SCENIHR) has also promoted a request for a scientific opinion on* the safety of surgical meshes used in urogynecological surgery*.

In consequence, the FDA is considering the approval of a new rule for the* Requirement for Premarket Approval for Surgical Mesh for Transvaginal Pelvic Organ Prolapse Repair*, in which the manufacturers should conduct animal studies to evaluate the* in vivo* performance of the meshes in an appropriate animal model. Undoubtedly, in situations in which transvaginal mesh is required, scientific preclinical data should support the specific medical devices. Hence, the present study using sheep as a preclinical animal model was performed to assess the outcome of n-butyl-2-cyanoacrylate (Histoacryl) as a fixation method for a light-weight and large pore size PP mesh (*synthetic PP Mesh-1*). At the same time, other mesh materials and fixation methods were used to compare the different outcomes and try to discern which factors were most directly related to a better host histocompatibility and lower incidence of graft related complications.

## 2. Materials and Methods

### 2.1. Animals

A total of six adult Lacaune (60–80 kg body weight) and 14 Ripollesa (35–70 kg body weight) ewes were used in this study. All animals were, unless stated, multiparous (2–5 pregnancies). Sheep were housed at Servei de Granges i Camps Experimentals, Universitat Autònoma de Barcelona. All sheep were kept at our facility for at least one week before surgery and were incorporated into the study after a physical examination as well as a hematological and biochemical blood test to detect any subclinical condition that could interfere in the experiment.

The animals were housed in individual boxes during a week after surgery and thereafter in groups. The diet consisted of pelleted diet and ad libitum hay. Tap water was supplied ad libitum. This study was conducted under protocols approved by the Ethical Committee of the UAB (1600) and Generalitat de Catalunya (6719).

### 2.2. Study Design

Three exploratory experiments were performed previously to the main experiment to compare the combination of PP mesh and Histoacryl with other materials, mesh sizes, and fixation methods. In all the pilot studies the follow-up time was three weeks, including a clinical monitoring and macroscopic and histologic score of the implantation sites after euthanasia. All studies and mesh materials assessed are summarized in Table 1.

A first pilot study was performed with the 6 Lacaune ewes to assess the clinical performance and histocompatibility of the* Synthetic PP Mesh-1* and Histoacryl as fixation method manually delivered through a 1 mL syringe (*PP-1-Hist* group). All other studies were performed with Ripollesa ewes. The rest of pilot studies served to define the experimental approach: mesh size and material, suture versus Histoacryl either manually or delivered by means of a specific device.

Once the results of the three pilot studies were analyzed, it was decided to perform the main study, which was a long-term study with 8 ewes assessing* Synthetic PP Mesh-1* in combination with Histoacryl using the reduced mesh size and a lower amount of Histoacryl. All ewes were implanted by posterior approach and distributed in two different follow-up periods (Table 1).

### 2.3. Implants and Fixation Methods

*Synthetic PP Mesh-1* and* Synthetic PP Mesh-2* (B. Braun Surgical, S.A) have a pore size between 2 and 2.5 mm type I mesh [9], a weight of 40 ± 5 g/m^2^ and a diameter thread of 0.125 ± 0.015 mm. Teflon mesh, Omyra mesh (CE marked by ProxyBiomedical), already used in clinical practice, consists of a micromachined cPTFE monolayer mesh with a density of 0.9 g/cm^2^ and a pore size of 2.4 mm. All meshes were sterilized with ethylene oxide and cut into patches of the required size for each study.

Monomeric n-butyl-2-cyanoacrylate surgical glue Histoacryl (B. Braun Surgical, S.A) already marketed for clinical practice was used by means of 1 mL syringe or* Histoacryl Pro-Set OFX* applicator.

The nonabsorbable monofilament polypropylene suture Premilene 2/0 was used as the anchoring system in the second and third pilot studies.

### 2.4. Surgery and Tissue Collection

Animals were premedicated with an intramuscular injection containing midazolam (Hospira Inc.) (0.2 mg/kg) and buprenorphine (Buprex Schering-Plough, S.A) (0.01 mg/kg). Anesthesia was induced with Propofol (B. Braun) (4 mg/kg) and the animals were intubated and maintained at a proper surgical anesthetic level through inhaled oxygenated Isoflurane (Isovet, B. Braun) (1.5–2.5%). A gastric catheter was placed into the stomach to avoid possible ruminal reflux during anesthesia.

**Figure 1:**
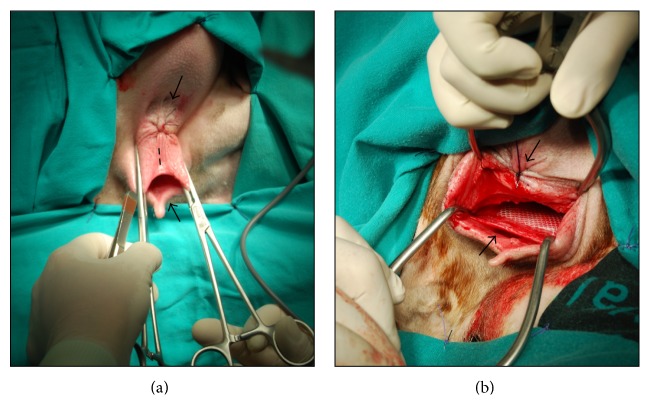
Photographs showing episiotomy procedure and posterior site mesh insertion (synthetic PP Mesh-1). (a) Dashed line shows the site where episiotomy with electric scalpel is performed. Arrows show the vaginal entrance and the anus. (b) Mesh placement on posterior site between the rectum and the dorsal vaginal wall.

For both approaches, anterior and posterior, an episiotomy was carried out (Figure 1). For the ewes implanted by an anterior approach, the urethra was catheterized with a Foley catheter and the vulva was incised in its ventral part, dissecting all the tissue between the urethra and the vaginal epithelium. The mesh was placed between the urine bladder and the vagina, attached to the ventral part of the vagina. For the ewes implanted in the posterior site, the vulva was incised in its dorsal part, dissecting the tissue to allow the fitting of the mesh between the vagina and the rectum (Figure 1). Then the mesh was fixed in the dorsal vaginal wall. The anterior implantation approach was surgically more difficult compared with the posterior approach. The proximity of the urethra to the implantation site and the narrow access led to a very high risk of urethral injury.

When the mesh was anchored with 2/0 polypropylene suture, a total of four stitches were made to secure it, one in each corner. When Histoacryl was used, the surgical glue was applied in each corner, with inner corners being the first to be anchored. In all cases, special care was taken to prevent the formation of folds in the mesh during implantation. After mesh placement, the vaginal wall was closed with a 2/0 synthetic short-term absorbable suture (Safil Quick).

All animals received an intravenous antibiotic dose of cefazoline (Kurgan, Normon S.A) just before starting the surgical procedure. Once the surgery was finished, an intramuscular dose of a long-acting antibiotic, ceftiofur 5 mg/kg (Naxcel, Pfizer, S.A), was administered. Analgesia was maintained for 10 days by means of subcutaneous meloxicam (Metacam, Boehringer Ingelheim, S.A) administration (0.3 mg/kg the day of surgery and 0.15 mg/kg for 10 days after surgery).

Sheep were euthanized at 3 weeks, 3 months, or 6 months, depending on the study. Euthanasia was performed by means of an overdose of sodium pentobarbital (Vetoquinol S.A) (60 mg/kg) given intravenously after a previous sedation. A necropsy followed by an internal examination of the thoracic and abdominal cavities was performed. The vagina was examined in order to assess the degree of fibrosis, possible mesh folding, infection, or vaginal erosion. Mesh implantation sites, including the mesh, the vagina, and rectum (opened longitudinally), were resected and fixed in a 4% formaldehyde solution altogether for histopathological assessment.

### 2.5. Clinical Evaluation

Animals were clinically monitored throughout the studies to assess possible discomfort and/or pain. For the first week after the surgery, animals were checked every day. The following week they were checked on alternate days, and on the third week they were monitored twice per week. For animals included in the 3- and 6-month follow-up study, they were monitored once a week from the fourth week onwards. Parameters monitored are displayed in Table 2.

Body weight gain was monitored in order to assess if the postsurgical period and subsequent mesh integration affected the body weight performance. Animals monitored for three weeks were weighed before surgery and at the end of the study. For animals included in the main study, weight control was performed once monthly.

### 2.6. Macroscopy

A semiquantitative score was applied during necropsy for all animals, including the assessment of the following parameters: fibrosis, presence of abscesses, mesh folding, vaginal exposure, intestinal exposure, and vaginal stricture (considered as obliteration in the vaginal lumen due to massive tissue ingrowth). Fibrosis was scored as 0 (none), 1 (slight), 2 (moderate), and 3 (intense). Abscess presence was scored as 0 (none), 1 (one abscess <0.5 cm), 2 (several abscesses <0.5 cm), and 3 (one or more abscesses >0.5 cm). The presence or absence of vaginal erosion, mesh folding, and vaginal stricture was assessed as percentage of prevalence in each group. This macroscopic score is the result of the assessment during necropsy and tissue trimming. During tissue trimming, the whole vagina with the mesh and the rectum were cut into several transversal slices and they were visually assessed to complete the necropsy score (Figures 3(a), 3(b), and 3(c)). Parameters like mesh folding were easily detected with these transversal cuts. Five slices were selected and processed into paraffin blocks, which were afterwards evaluated histologically.

### 2.7. Histopathology

2 *μ*m thick slides were cut for each of the 5 blocks processed from each animal. The slides were stained with hematoxylin-eosin and evaluated by a pathologist. Digital images were obtained with a light microscope (*Leica DM 6000B*) coupled with a digital camera (*Leica DFC480*; Leica Microsystems CMS GmBh, Germany). A semiquantitative histological score for each of the slides was performed and the mean score of the five preparations was calculated.

### 2.8. Statistical Analysis

All statistical analysis was performed using GraphPad Prism 4 (GraphPad Software, La Jolla, CA, USA). Data are expressed as mean ± SEM. Histological scoring results from the main study group were compared with the results of the* PP-1-Hist posterior* group of the first pilot study by means of paired *t*-test with 95% of confidence interval. Histological results from the two different periods of follow-up in the main study were also compared amongst them.

## 3. Results

### 3.1. Clinical Evaluation

Overall, the daily score of clinical signs was low in all groups of animals, not exceeding 1.5 points out of 21 (Table 3). Clinical signs were mainly related to wound appearance, which lasted generally until the wound was totally healed. The episiotomy wound underwent a normal healing process in all animals without finding any specific complications. The highest clinical symptoms were found in the pilot studies, specifically in the animals which were implanted anteriorly with PP mesh anchored with PP stitches. Apart from apathy and decrease of food ingestion in some of the affected animals, the rest of the clinical signs monitored throughout the studies were absent during the experimental periods. Overall, the clinical score was higher in animals in which mesh vaginal exposure was detected macroscopically or microscopically. Apathy including unwillingness to stand up was observed in the first pilot study, within the* PP-1-Hist posterior* group, where one of the ewes suffered an anus-vaginal fistula. In this case, additional analgesic treatment was supplied by means of subcutaneous buprenorphine. There were no significant differences in body weight gain amongst the studied groups.

### 3.2. Macroscopy

**Figure 2:**
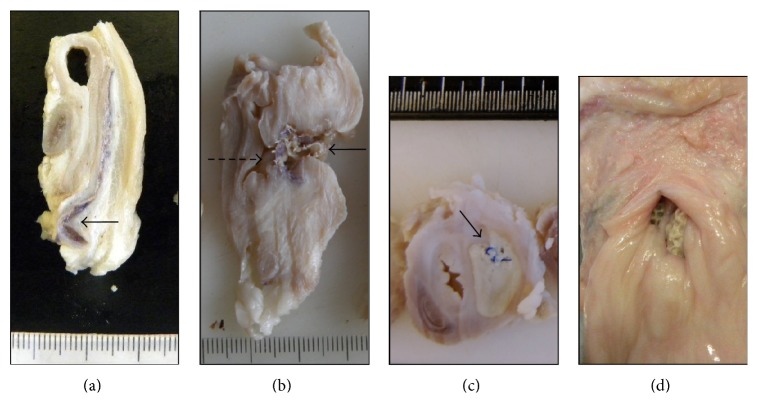
Photographs showing some of the macroscopic findings described during tissue trimming. (a) PP-1-Hist-6 month group. Note the good position of the mesh despite the slight folding in one of the corners, which was the unique macroscopic finding observed in this group. (b) PP-1-Hist posterior 3 wk group. Note the complete folding of the mesh with exposure of the mesh into the vaginal cavity (dashed arrow) and into the colonic cavity (continuous arrow). (c) PP-2-PP posterior. Abscess associated to one of the polypropylene stitches. (d) PP-1-PP anterior 3 wk. Extrusion of the mesh into the vagina.

The main macroscopic findings are shown in Table 3 and Figure 2. Overall, the two groups belonging to the main study with* Synthetic PP Mesh-1* in combination with device applied Histoacryl displayed less mesh exposures, mesh folding, and abscesses. There was only one animal in which the mesh was partially folded in one corner, without causing further complications (Figure 2(a)).

### 3.3. Histopathology

**Figure 3:**
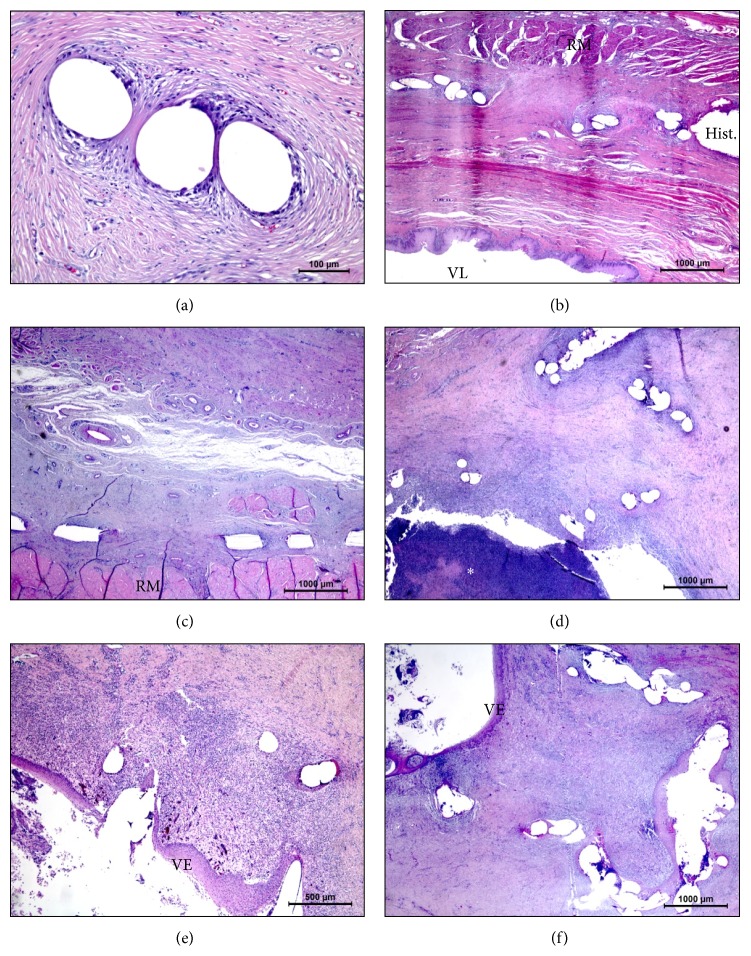
Histological hematoxylin-eosin stained sections of the different implants. (a)* PP-1-Hist-6m* group showing good integration of the PP mesh with slight foreign body reaction. (b)* PP-1-Hist-3m* group showing good integration of the mesh and Histoacryl (Hist.) with flattened position and good location versus vaginal lumen (VL) and rectal musculature (RM). (c)* Omy-TF-PP posterior* group showing proper location of the mesh and good integration. (d)* PP-1-Hist anterior* group showing a purulent reaction (asterisk) and folding of the mesh. (e)* PP-1-PP anterior* group showing folding of the mesh and vaginal inflammation and erosion with vaginal epithelium (VE) ulceration. (f)* PP-2-PP posterior* group showing vaginal inflammation and ulceration due to mesh exposure.

The best implant integration within the tissue was observed in the main study (synthetic PP mesh-1 and device applied Histoacryl) as well as in the pilot study with cPTFE mesh (Table 4). The inflammatory reaction was also very low in the main study, being almost nonexistent in the explants examined at 6 months postimplantation. In these 8 animals, there were no cases of vaginal ulceration or even vaginal epithelial inflammation. The mesh was evenly distributed and situated between the rectal musculature and the dorsal vaginal wall, displaying a solid bridging fibrosis (Figure 3(b)) which had also significantly higher scores of mesh integration with respect to the first pilot study (*P* < 0.05).

## 4. Discussion 

The aim of this study was to find a proper experimental approach and assess the performance of a low-weight PP mesh with large pore (≥2 mm) anchored with surgical glue (Histoacryl) in pelvic floor surgery in a sheep model. After 3 and 6 months of follow-up, the tissue integration was good together with an inexistent rate of graft-related complications (GRCs). The comparison of this suitable combination with other synthetic mesh materials and fixation methods within the study shows that a proper mesh size and a good fixation method to avoid folding and mesh exposures are key features for a good performance of pelvic floor implanted synthetic meshes.

The sheep has been explored in recent years as a preclinical animal model in pelvic floor disorders [10, 11]. Its good behavior, inexpensiveness, and similarities with women in terms of predisposition to suffer from POP make sheep a suitable model. Despite being a quadruped animal, it can help predict the behavior of biologic/synthetic graft materials used in pelvic floor reconstruction, providing information related to postoperative pain, as seen in the present study. Indeed, this is the first preclinical study correlating mesh histocompatibility with clinical symptoms in sheep.

One of the most serious complications resulting from pelvic floor synthetic mesh implantation in women is the occurrence of dyspareunia, which is normally related to mesh exposure into the vagina [6, 7]. The assessment of clinical parameters such as food intake, animal posture, or wound appearance has allowed us to obtain a clinical score which correlates to a large extent with the outcome of the implanted grafts. The highest clinical scores were obtained for the meshes implanted between the urinary bladder and the vagina (anterior site), which has been also proven to be a location more liable to complications in women [12]. Perfecting this clinical assessment together with the development of a semiquantitative system for assessing local pain would help obtain very valuable data in these preclinical studies.

Vaginal mesh exposure or even extrusion of the mesh into the vagina is the main complication in pelvic floor reconstruction with synthetic meshes. Thus, nowadays, an effort for standardization of symptoms and severity related to POP complications is being made in order to address the best treatment approach for each patient [13]. As previously reported [14], mesh exposures were detected in our study. The highest mesh exposure rate was of 66% of animals in the first pilot study. As suggested by [15], a big mesh size could trigger the presence of graft-related complications (GRCs). This fact might be one of the reasons of the high prevalence of vaginal mesh extrusion. In fact, the reduction of mesh size in the long-term study leads to better results. In our case, the use of an excessive amount of surgical glue (Histoacryl) might be also helping in the high prevalence of vaginal mesh extrusion. At the same time, mesh exposure/extrusion clearly correlated with the presence of mesh folding, a fact that has also been observed clinically [7].

Good mesh fixation is crucial to avoid complications. Mesh stiffness has been pointed out as a factor directly related to the avoidance of mesh folding [15, 16]. The combination of the condensed polytetrafluoroethylene Omyra mesh with PP suture was the only combination tested in our pilot studies which obtained integration and inflammatory results as good as those obtained with the PP Mesh-1 with Histoacryl. The high stiffness offered by the Omyra cPTFE mesh could be the reason for this good performance, allowing a flat position with avoidance of folds and leading to a good integration.

Polypropylene is known as an inert synthetic material with good integration, being the most used synthetic graft material. Nowadays, the low-weight PP meshes with large pore size are the first choice because they allow a better tissue ingrowth with better graft integration [17, 18]. However, these meshes are very soft and prone to folds. When combining the PP Mesh-1 with Histoacryl as a fixation method, the glue polymerization provides the PP mesh with a degree of stiffness that helps achieve a good position and avoidance of folds. This n-butyl-2-cyanoacrylate synthetic glue has been extensively used for wound closure and has recently been proven to be a good alternative method for mesh fixation in hernias [19–21]. In our study, for the first time, we assessed* in vivo* the outcome of n-butyl-2-cyanocrylate as fixation method for synthetic mesh placement in pelvic floor surgery using the sheep as a model. In our experience, when the glue was applied manually by means of a syringe, it was difficult to control the amount of glue placed and, in consequence, large volumes of surgical glue led to a pyogranulomatous foreign body inflammation which was hard to reabsorb and caused mesh folding, exposure, and/or infection as seen in the first pilot study. Moreover, large amounts of glue may be impairing tissue ingrowth through the mesh pores, leading to poor tissue integration. In contrast, the use of* Histoacryl Pro-Set OFX* applicator allowed us to control the appropriate volume of adhesive which conferred the proper level of strength for fixation allowing a good integration of the surrounding tissue. No complications or side effects were observed in the animals after 3 and 6 months of follow-up, with the mesh being well fixed and integrated in all cases.

In summary, our study demonstrates that the use of light weight* Polypropylene-1 mesh* in combination with n-butyl-2-cyanocrylate glue (*Histoacryl*) applied with* Histoacryl Pro-Set OFX* applicator in sheep does not induce any graft-related complications in pelvic floor implantation and provides a good histological integration.

## Figures and Tables

**Table 1:** Summary of the different experimental groups showing mesh material and anchorage characteristics.

Experiment	Experimental group	(*n*) Surgical approach	Type of mesh	Anchorage used	Mesh size (width-length)	Follow-up period
1	PP-1-Hist	3 anterior	Synthetic PP Mesh-1	Histoacryl 0.4–0.8 mL	5 × 5 cm	3 weeks
		3 posterior				

2	PP-1-PP	1 anterior	Synthetic PP Mesh-1	PP 2/0 Premilene	3 × 4 cm	3 weeks
		1 posterior			4 × 4 cm	

3	Omy-TF-PP	2 posterior	cPTFE Omyra Mesh	PP 2/0 Premilene	2 × 2 cm	3 weeks
					3 × 3 cm	
	PP-2-PP	1 anterior	Synthetic PP Mesh 2	PP 2/0 Premilene	2 × 2 cm	3 weeks
		1 posterior			3 × 3 cm	

4	PP-1-Hist-3m	4 posterior	Synthetic PP Mesh-1	Histoacryl 0.15–0.25 mL	3 × 3.2 to 3.5 × 3.5 cm	3 months
	PP-1-Hist-6m	4 posterior	Synthetic PP Mesh-1	Histoacryl 0.15–0.25 mL	3 × 3.2 to 3.5 × 3.5 cm	6 months

PP: Polypropylene; cPTFE: condensed polytetrafluoroethylene.

**Table 2:** Clinical signs monitored during *in vivo* study.

Parameter	Score
Claudication	None = 0; mild = 1; moderate = 2; severe = 3
Posture	None = 0; it stands up without forcing but with difficult = 1; it needs to be forced to stand up = 2; does not stand up even being forced = 3
Mucous coloration	Pink = 0; pale = 1; cyanotic = 3
Corporal temperature	Fever: none = 0; mild = 1; severe = 3
Wound appearance	Inflammation: none = 0; mild = 1; moderate = 2; severe = 3
Food ingestion	Decrease on ingestion: none = 0; mild = 1; moderate = 2; complete anorexia = 3
Feces/urine	Normal = 0; abnormal = 3
Total score	21

**Table 3:** Data showing clinical and macroscopic scores in pilot study groups and main study groups (bold). Note that mean daily clinical score is based on 21 days for pilot studies and on 3 and 6 months in the definitive study.

Experimental group	Mean daily clinical score (up to 21 points)	Macroscopic score					
		Fibrous reaction	Abscesses	Vaginal exposure	Intestinal exposure	Mesh folding	Vaginal stricture
PP-1-Hist anterior (5 × 5 cm)	0.42	2.33	1	2/3	0/3	2/3	0/3
PP-1-Hist posterior (5 × 5 cm)	0.60	2.67	1^b^	2/3	1/3	1/3	0/3
PP-1-PP anterior (3 × 4 cm)	1.00	2	0	1/1	0/1	1/1	0/1
PP-1-PP posterior (4 × 4 cm)	0.77	3	1^b^	1/1	0/1	1/1	0/1
Omy-TF-PP posterior (2-3 × 3-3 cm)	0.63	1.5	0	0/2	0/2	0/2	0/2
PP-2-PP anterior (2 × 2 cm)	0.85	2	0	0/1	0/1	0/1	0/1
PP-2-PP posterior (3 × 3 cm)	0.46	3	3	1/1	0/1	1/1	0/1
**PP-1-Hist-3m (3–3.5 × 3.2–3.5)**	**0.42**	**1.75**	**0/4 **	**0/4 **	**0/4 **	**0/4 **	**0/4 **
**PP-1-Hist-6m (3–3.5 × 3.2–3.5)**	**0.28**	**1**	**0/4 **	**0/4 **	**0/4 **	**1/4** ^a^	**0/4 **

^a^Fold in one of the corners of the mesh. It does not represent the complete folding of the mesh.

^b^Abscesses detected by histology of approximately 5 mm of diameter but considered as macroscopic finding.

**Table 4:** Histological results of the pilot studies and the final study (bold).

*N*	Experimental group	Fibrosis	Mesh integration	Inflammatory reaction	Inflammatory cell type	Vaginal mucosal inflammation	Vaginal epithelium ulceration	Epithelial cicatrisation^*∗*^
*n* = 3	PP-1-Hist anterior	2 ± 0.38	1.3 ± 0.14	2.4 ± 0.06	2.5 ± 0.19	0.8 ± 0.23	0.6 ± 0.18	0
*n* = 3	PP-1-Hist posterior	1.3 ± 0.10	1.4 ± 0.04	2.1 ± 0.23	2.2 ± 0.24	1.2 ± 0.18	0.9 ± 0.17	1 ± 0.38
*n* = 1	PP-1-PP anterior	1.6	1	1.8	2.6	1.8	1	0
*n* = 1	PP-1-PP posterior	0.4	0	1.4	2.2	3	2	0
*n* = 2	Omy-TF-PP posterior	2.8 ± 0.14	3 ± 0	1 ± 0	1.3 ± 0.07	0	0	—
*n* = 1	PP-2-PP anterior	2	2	0.8	1.2	0	0	—
*n* = 1	PP-2-PP posterior	0.8	0	1.8	2.2	1.2	0.8	0
**n** = 4	**PP-1-Hist-3m**	**2.5 ± 0.09** ^a^	**3 ± 0** ^b^	**1 ± 0** ^c^	**1 ± 0.04**	**0**	**0** ^c^	**—**
**n** = 4	**PP-1-Hist-6m**	**2.5 ± 0.14**	**3 ± 0** ^b^	**0.1 ± 0.03**	**1 ± 0.02**	**0**	**0**	**—**

Inflammatory reaction (0 = none; 1 = slight; 2 = moderate; 3 = intense).

Inflammatory cell type (0 = without inflammatory cells; 1 = macrophagic; 2 = lymphocitic; 3 = neutrophilic).

Fibrosis (none = 0; slight = 1; moderate = 2; intense = 3).

Mesh integration (encapsulation, without integration = 0; poor integration = 1; deficient integration = 2; good integration = 3).

Vaginal epithelium inflammation (none = 0; macrophagic inflammation = 1; lymphocytic inflammation = 2; neutrophilic inflammation = 3).

Vaginal epithelium ulceration (none = 0; slight = 1; moderate = 2; intense = 3).

Vaginal epithelium cicatrisation (absence of epithelization = 0; slight epithelization = 1; moderate epithelization = 2; good regeneration and epithelization = 3).

^*∗*^Cicatrisation is only assessed when “vaginal ulceration” reaches at least a score of ≥1. *N* = 1–4. Data for each animal is the mean value obtained from the evaluation of 5 hematoxylin-eosin slides.

^a^ *P* < 0.05 versus PP-1-Hist posterior; ^b^ *P* < 0.01 versus PP-1-Hist posterior, ^c^ *P* < 0.01 versus PP-1-Hist-6m.
